# Mechanisms of TP53 Pathway Inactivation in Embryonic and Somatic Cells—Relevance for Understanding (Germ Cell) Tumorigenesis

**DOI:** 10.3390/ijms22105377

**Published:** 2021-05-20

**Authors:** Dennis M. Timmerman, Tessa L. Remmers, Sanne Hillenius, Leendert H. J. Looijenga

**Affiliations:** Princess Máxima Center for Pediatric Oncology, Heidelberglaan 25, 3584 CS Utrecht, The Netherlands; D.M.Timmerman-6@prinsesmaximacentrum.nl (D.M.T.); tessa.l.remmers@gmail.com (T.L.R.); hillenius.sanne@gmail.com (S.H.)

**Keywords:** P53 pathway, cancers, mutations, embryonic and somatic cells

## Abstract

The P53 pathway is the most important cellular pathway to maintain genomic and cellular integrity, both in embryonic and non-embryonic cells. Stress signals induce its activation, initiating autophagy or cell cycle arrest to enable DNA repair. The persistence of these signals causes either senescence or apoptosis. Over 50% of all solid tumors harbor mutations in *TP53* that inactivate the pathway. The remaining cancers are suggested to harbor mutations in genes that regulate the P53 pathway such as its inhibitors Mouse Double Minute 2 and 4 (MDM2 and MDM4, respectively). Many reviews have already been dedicated to P53, MDM2, and MDM4, while this review additionally focuses on the other factors that can deregulate P53 signaling. We discuss that P14^ARF^ (ARF) functions as a negative regulator of MDM2, explaining the frequent loss of ARF detected in cancers. The long non-coding RNA Antisense Non-coding RNA in the *INK4* Locus (ANRIL) is encoded on the same locus as *ARF*, inhibiting ARF expression, thus contributing to the process of tumorigenesis. Mutations in tripartite motif (TRIM) proteins deregulate P53 signaling through their ubiquitin ligase activity. Several microRNAs (miRNAs) inactivate the P53 pathway through inhibition of translation. CCCTC-binding factor (CTCF) maintains an open chromatin structure at the *TP53* locus, explaining its inactivation of CTCF during tumorigenesis. P21, a downstream effector of P53, has been found to be deregulated in different tumor types. This review provides a comprehensive overview of these factors that are known to deregulate the P53 pathway in both somatic and embryonic cells, as well as their malignant counterparts (i.e., somatic and germ cell tumors). It provides insights into which aspects still need to be unraveled to grasp their contribution to tumorigenesis, putatively leading to novel targets for effective cancer therapies.

## 1. Introduction

### 1.1. Embryonic Stem Cells and Germ Cell Tumors Versus Somatic Cells

**Figure 1:**
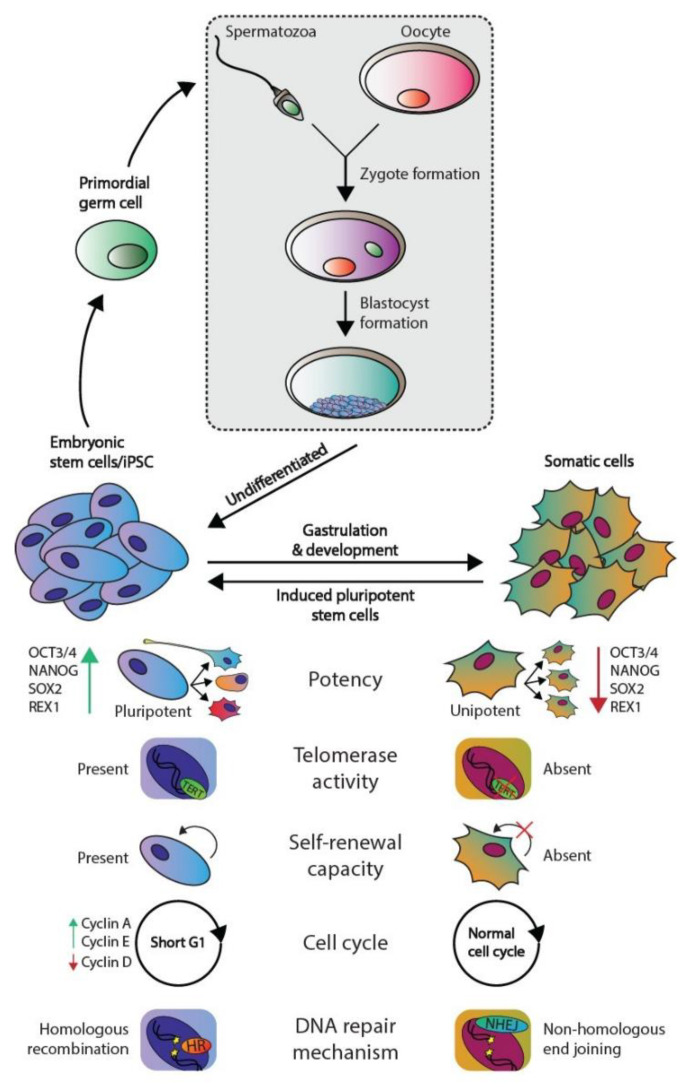
Phenotypic differences between embryonic and somatic cells. Fertilization of an oocyte by a spermatozoon forms a zygote, which subsequently develops the blastocyst. The blastocyst contains embryonic stem (ES) cells that eventually give rise to all somatic cell types. ES and somatic cells can be distinguished according to multiple aspects: their potency, telomerase activity, self-renewal capacity, cell cycle regulation, and preferred DNA repair mechanism. Both germ cells and germ cells tumors are derived from ES derived PGCs and therefore harbor the same embryonal characteristics.

The development of multicellular, sexually reproductive organisms starts with the fusion of a spermatozoa and an oocyte that created a diploid zygote (depicted in Figure 1). Then, the zygote divides, forming a cluster of undifferentiated cells (i.e., blastomeres) known as the morula. The first step of lineage differentiation occurs during the subsequent blastocyst stage in which the embryoblast (i.e., the inner cell mass) and the trophectoderm are formed [1,2]. The trophectoderm develops into all extra-embryonic structures, whereas the embryoblast, which consists of pluripotent embryonic stem (ES) cells, develops into all embryonic structures [2]. ES cells are transiently present, can self-renew and give rise to all three embryonic germ layers during gastrulation (i.e., the ecto-, meso-, and endoderm), which ultimately will give rise to all cell lineages of the adult organism [2]. Additionally, ES cells give rise to the primordial germ cells (PGC) in the embryonic yolk sac, which subsequently migrate toward developing gonads to give rise to the germ line [2,3] (see Figure 1).

The pluripotent nature of ES cells is characterized by the expression of pluripotency markers including but not limited to OCT4, NANOG, SOX2, and REX1 (Figure 1) [4]. In contrast, somatic cells are differentiated and thus lineage-restricted and unipotent [5]. Therefore, these cells seldomly give rise to other progeny than the identity of the cells themselves [5]. In addition, due to their capacity to self-renew, ES cells can be cultured indefinitely in vitro while retaining their embryonic state as well as a stable genome. This immortality is facilitated by active telomerase, which is an enzyme that extends telomeric repeats that are otherwise lost due to the end-replication problem after each cell cycle (approximately 50–150 base pairs are lost per cycle) [6,7] (Figure 1). Conversely, somatic cells have a restricted lifespan due to the lack of active telomerase and induce cellular senescence when a critical telomere length is reached [6,7]. Furthermore, ES cells also regulate the cell cycle differently as these lack Cyclin D expression yet continuously express Cyclin A and E, which leads to a significantly shortened G_1_-phase when compared to somatic cells [8]. This characteristic also facilitates the tendency for ES cells to initiate apoptosis rather than cell cycle arrest and DNA repair when DNA damage occurs, the latter of which occurs predominantly in the G_1_ phase after cell cycle arrest [9,10]. The preference for apoptosis in these early embryonic cells is considered a failsafe mechanism to preserve the genetic integrity of their multitudinous progeny. In rare occasions, ES cells can initiate DNA repair; however, these cells then opt for error-free homologous recombination (HR) to repair double-strand breaks, whereas somatic cells predominantly employ error-prone non-homologous end joining (NHEJ) [9]. Finally, a zygote is known to lose its inherited DNA methylation pattern immediately after fertilization and subsequent changes in DNA methylation occur during early embryonic development as various cell lineages arise. Thus, DNA methylation patterns between ES and somatic cells vary significantly [11,12]. 

Of note, a discovery made over a decade ago demonstrated that differentiated somatic human cells (e.g., adult human fibroblasts) could be reprogrammed to a pluripotent state reminiscent of ES cells (i.e., nuclear reprogramming) [13]. These cells are known as induced pluripotent stem cells (iPSCs) and, in theory, they can also be maintained in a pluripotent state indefinitely. iPSCs are originally generated in vitro by overexpressing a cocktail of four essential genes (*OCT3/4*, *c-MYC*, *SOX2*, and *KLF4*) in the differentiated cells [14].

Moreover, many of the characteristics of ES cells are strongly conserved in germ cell tumors (GCTs), which represent a heterogeneous cluster of solid tumors that originate from both ES cells and PGCs [3]. Of note, the parallels observed between ES cells and GCTs do not apply to the teratomas, which is a GCT subtype that consists of fully differentiated cells and harbor significant differences in their (epi)genetic makeup and clinical response (i.e., inherent chemotherapeutic resistance) compared to other GCT subtypes [3,15,16,17]. An example of the similarities observed between ES cells and GCTs is that the latter also express multiple pluripotency markers including OCT3/4 and NANOG and have active telomerase activity, again with the exception of teratoma [3]. Moreover, it is widely accepted that in response to DNA damage, GCTs also favor apoptosis, which is thought to underlie their unique sensitivity to platinum-based chemotherapeutics, including cisplatin (a key component of GCT treatment) [9,10,18]. This is supported by additional evidence demonstrating that GCTs have low expression of genes involved in cell cycle arrest and a deregulated G1-S phase checkpoint, thus potentially also preventing the activation of DNA repair pathways [18,19,20,21,22,23]. Moreover, the apoptotic response of ES cells and GCTs in response to DNA damage has been attributed to the well-known P53 pathway; however, at least in GCTs, it remains a topic of debate [18,21,24,25,26,27,28,29,30,31,32,33,34,35,36,37]. The P53 pathway has been dubbed the guardian of the genome due to its integral role in maintaining genomic integrity among all cell types. This pathway acts in response to cellular stress and lies at the apex of a plethora of downstream signaling pathways including cell cycle arrest, DNA repair, and apoptosis. The deregulation of this pathway has been observed in cancer and is strongly associated to many aspects of tumorigenesis. For instance, GCTs most often express high levels of wild-type (WT) *TP53*, and it has been suggested that the P53 pathway is (partially) responsible for their characteristic to enter apoptosis in response to platinum-based chemotherapeutics (e.g., cisplatin) which is reminiscent of ES cells in response to DNA damage [10,18,21,23,25,26,27,28,29,33,34,35,36,37]. In addition, it has been suggested that the P53 pathway is deregulated through multiple mechanisms in GCTs that have acquired resistance to chemotherapy, including cisplatin [15,18,38,39,40,41,42]. 

In short, the importance of the P53 pathway and its differential regulation between somatic and ES cells (and in parallel GCTs, except teratoma) underlines the purpose of this review, which is to provide an up-to-date overview of the important roles, regulators, and downstream effectors of the P53 pathway. In addition, multiple mechanisms that are known to inactivate this pathway and contribute to tumorigenesis will be highlighted. 

### 1.2. Protecting Genomic Integrity

Genomic changes during development serve as the driving force of evolution; however, these may also negatively impact multiple cell lineages with possible detrimental effects on both the individual and their offspring. Therefore, protection of the genomic integrity is paramount for a species to survive and commences during early embryonic development [23]. For example, the oocyte expresses and deposits multiple mRNAs that protect the zygote against DNA damage during the first cellular divisions [43]. As mentioned before, there have been several indications of a robust protective mechanism in ES cells including the removal of mutated ES cells from the stem cell pool through apoptosis, which is a characteristic that is seemingly conserved in GCTs [7,12,44]. Alternatively, ES cells may also be instructed to differentiate through transcriptional inhibition of the pluripotency factor *NANOG*, which also effectively removes ES cells from the stem cell pool [45]. These mechanisms in combination with lacking a G_1_ checkpoint are thought to result in a 100-fold lower frequency of accumulating mutations in at least murine ES cells compared to mouse embryonic fibroblasts, which resemble the somatic cells [9,23]. Conversely to ES cells and GCTs, somatic cells utilize different pathways (DNA repair rather than apoptosis) to protect their genome that enable these to survive while risking an increased mutational load [9,23].

### 1.3. P53 Pathway

**Figure 2:**
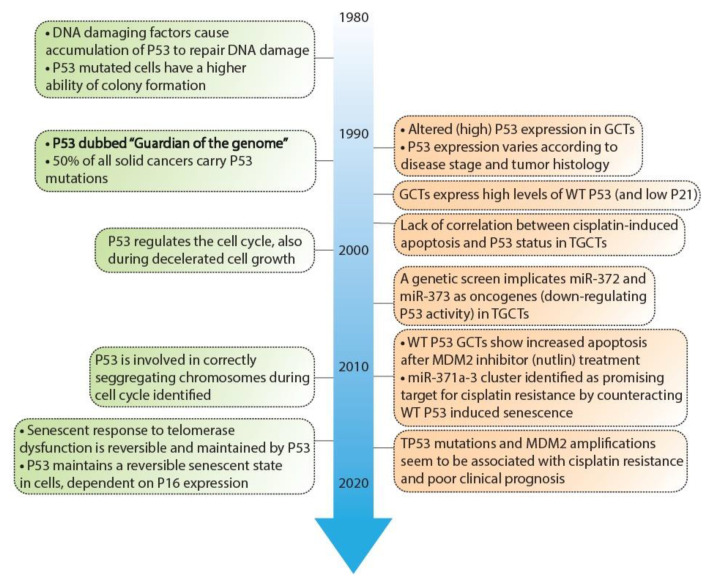
Timeline depicting key discoveries in P53 signaling and germ cell tumors. A timeline of the discoveries regarding P53 as a guardian of the genome (green) and studies regarding TP53 in GCT development and resistance (orange). References key discoveries P53 (green): 49–58, references key discoveries TP53 in GCT development (orange): 25–41, 59–62.

**Figure 3:**
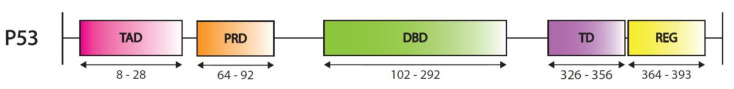
Schematic overview of the P53 protein. Indicated are (from N- to T-terminus) TAD: Transactivation domain, PRD: Proline-rich domain, DBD: DNA-binding domain, TD: Tetramerization domain and REG: Regulatory domain. Numbers indicate the amino acids included in each domain.

As mentioned before, the P53 pathway is essential in maintaining genomic integrity. Central to this pathway is the P53 protein that is regarded as a tumor suppressor and originates from the *TP53* gene. Several key discoveries regarding P53 are depicted in Figure 2, as well as its relevance in GCTs [22,23,24,25,26,27,28,29,30,31,32,33,34,35,36,37,38,46,47,48,49,50,51,52,53,54,55,56,57,58,59]. In response to cellular stress, the P53 protein is activated and accumulates in the cell after which it mainly functions as a transcription factor that transactivates a plethora of downstream targets [49]. These target genes are key players in one of many downstream pathways including cell cycle arrest, DNA repair, apoptosis, senescence, and autophagy [51,60,61]. The P53 protein counts 393 amino acids (AA) and consists of multiple domains, starting with a transactivation domain (TAD) at the amino-terminus (N-terminus), a directly neighboring proline-rich domain (PRD), a large DNA-binding domain (DBD), a tetramerization domain (TD), and a carboxy-terminus (C-terminus) regulatory domain (REG) [62,63] (depicted in Figure 3). Once activated and accumulated in the cell, the P53 protein functions and binds DNA as a tetramer, which is facilitated by the TD [64]. When tetramerized, the DBD contains three loops, L1, L2, and L3 respectively [65]. Both L2 and L3 are bound by a zinc ion, effectively linking both loops and enabling L3 to bind to the minor groove of the DNA, while a helix in the DBD is complementary to the major groove [65,66]. 

**Figure 4:**
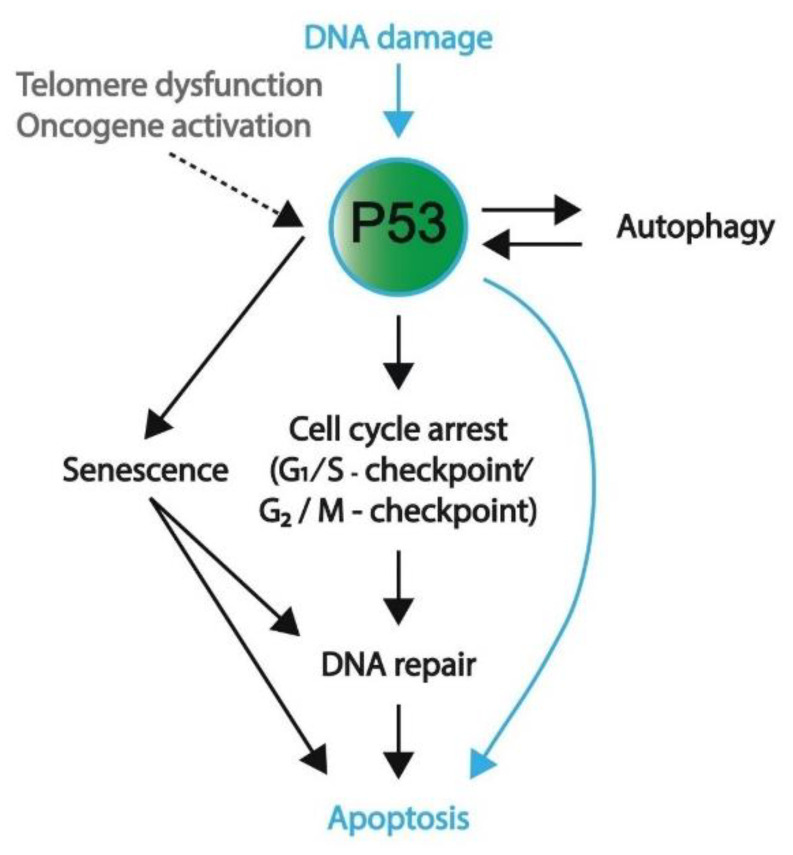
A schematic overview representing the favored mechanism of P53 activation in embryonic cells and somatic cells. DNA damage, both in embryonic (blue) and somatic (black) cells, will induce P53 activation. To protect genomic integrity, embryonic cells favor apoptosis over DNA repair. Conversely, somatic cells preferentially engage in cell cycle arrest and DNA repair; however, if DNA damage persists, these cells will enter apoptosis or become senescent. Furthermore, telomere dysfunction and oncogene activation are other means to activate P53 signaling (mostly in somatic cells). In addition to apoptosis, P53 activation and its downstream pathways can result in autophagy, senescence, and cell cycle arrest.

There are many different forms of cellular stress such as DNA damage that activate upstream regulators of p53, leading to the activation of the p53 pathway. For example, Ataxia-telangiectasia mutated (ATM) kinase serves as an important DNA damage sensor, as it recognizes and binds to double-strand DNA breaks and subsequently activates the P53 pathway, ultimately resulting in the activation of NHEJ or HR DNA repair mechanisms [55,67]. ATM phosphorylates histone H2AX, which is a variant of histone H2A that recruits molecules responsible for H3K9 methylation [55]. In turn, methylated H3K9 leads to the acetylation and activation of ATM [55]. Among other substrates, ATM phosphorylates and activates Checkpoint Kinase 1 and 2 (CHK1 and CHK2, respectively) [55]. In turn, CHK1 and 2 function as transcriptional activators by phosphorylating and activating the P53 protein [68]. The subsequent section will further outline several downstream targets and their related pathways including cell cycle arrest, senescence, apoptosis, and autophagy (also summarized in Figure 4).

#### 1.3.1. Induction of Cell Cycle Arrest

As indicated, DNA damage leads to the accumulation of P53, which induces cell cycle arrest, and subsequent DNA repair cell cycle arrest can occur at both the G1/S and G2/M checkpoints and is mostly effectuated by P21 (*CDKN1A)*, which is a target of P53 [69]. Activated P53 binds to the promotor of P21 and initiates its transcription [70,71]. P21 inhibits the function of cyclin-dependent kinases (CDKs) CDK2 and CDK4/6 present at the G_1_/S transition, which results in the hypomethylation of pRB-related proteins p107 and p130 [69,72]. The transcription of cell cycle-promoting genes will subsequently be repressed, inducing a temporary block of cell cycle arrest that enables the cell to initiate DNA repair mechanisms.

#### 1.3.2. Senescence or Apoptosis as a Final Measure to Protect Genomic Integrity

Chronic cellular stress signals such as telomere dysfunction, persisting DNA damage, and oncogene activation result in prolonged P53 activation and subsequent P21 expression, which in turn may induce cellular senescence or apoptosis (the latter of which is the main outcome in ES cells and GCTs) [72]. In contrast to cell cycle arrest, senescence is a stable state of arrest, and both processes are initiated by P53, again illustrating the tumor-suppressive functions of this protein [55,72]. Conversely, a senescent cell is able to re-enter the cell cycle after loss of P53 and has been shown to contribute to tumorigenesis, as it results in fast cell proliferation [27,28,73]. Prolonged P21 expression eventually induces the expression of P16^INK16A^ (hereafter referred to as P16), which similarly functions as a CDK inhibitor for CDK4/6, thereby indirectly inhibiting pRb-family members [72]. P16 expression is known to be regulated by epigenetic factors including DNMT3, which facilitates de novo methylation of the P16 promotor, inhibiting its expression, which is maintained by DNMT1 [55]. Demethylation of the promotor consequently induces P16 expression [55]. In addition to P16, senescence is also regulated by P53-independent pathways including NF-κb, which is responsible for the expression of proinflammatory cytokines that are secreted during senescence/this cellular state [55,72]. Notably, senescent cells are non-responsive to apoptotic signals, which is demonstrated by the continued activation of cAMP response element-binding (CREB), which functions as a transcription factor for the anti-apoptotic BCL-2 protein [55]. Alternatively, persisting cellular stress signals may induce apoptotic pathways, and further information of these pathways is described in Box 1. 

#### 1.3.3. Autophagy

**Figure 5:**
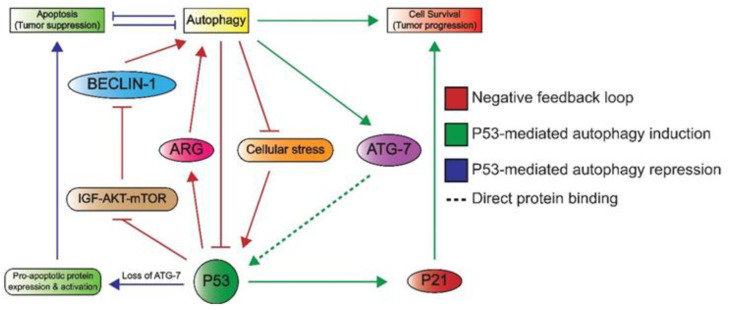
An overview of the crosstalk between P53 and autophagy. Autophagy can repress P53 through the inhibition of upstream activating proteins as well as P53 itself. This forms a negative feedback loop (red) as P53 induces autophagy through the inhibition of the IGF–AKT–mTOR pathway and subsequent increase in BECLIN-1 levels, which plays a role in autophagosome formation. P53 also induces the expression of autophagy-related genes (ARG). Autophagy induces cell survival, which is effectuated by the binding of ATG7 to P53, ultimately leading to P53-dependent P21 expression and subsequent cell cycle arrest (green). The loss of ATG-7 results in apoptosis (blue), as P53 induces the expression and activation of pro-apoptotic proteins. Apoptosis and autophagy have mutually inhibitory effects on one another.

Lastly, P53 can induce autophagy, which underlines its additional role in cellular metabolism. Autophagy enables cells to maintain homeostasis by the removal and recycling of (faulty) organelles [74]. Recycling results in an increase of anabolic intermediates that can subsequently be used in multiple pathways [74,75]. There are many connections between P53 and autophagy, as illustrated in Figure 5, as autophagy is able to inactivate P53 through different mechanisms such as proteosomal degradation [76]. In addition, the autophagy protein ATG7 has been reported to bind P53 and activate the transcription of P21, resulting in cell cycle arrest [74]. Conversely, P53 promotes the transcription of genes involved in autophagy, which suggests a negative feedback loop between the autophagy proteins and P53 [74]. Additionally, a more indirect crosstalk between p53 and autophagy via the mTOR pathway is known to occur in multiple tissue types including skeletal muscle, heart, white fat, liver, and kidney tissue [33]. Here, P53 promotes the transcription of negative regulators of the IGF–AKT–mTOR pathway, resulting in higher levels of BECLIN-1, which is part of the phosphatidyl inositol-3 kinase (PI3K) complex and plays an important role in the formation of autophagosomes [77,78]. Notably, a loss of BECLIN-1 results in an increased risk of tumorigenesis, which underlines its importance in autophagy and maintaining genomic integrity [78].

Box 1 **The P53-mediated mitochondrial apoptotic pathway.**

Under unstressed conditions, apoptosis is inhibited by anti-apoptotic proteins BCL-2, BCL-X_L_, BCL-W, MCL1, and A1, which interact with and inhibit pro-apoptotic proteins such as BAX and BAK [79]. These pro- and anti-apoptotic proteins are members of the BCL-2 family and contain up to four BCL-2 homology (BH) domains [79,80]. BH3-only proteins contain only the BH3 domain and are pro-apoptotic proteins within the BCL-2 family [81]. When DNA damage persists, P53 activation results in the expression of pro-apoptotic protein BAX and BH3-only proteins PUMA and NOXA. The latter two in combination with P53 induce apoptosis by releasing BAX and BAK from their inhibitory binding with various anti-apoptotic proteins [81,82,83]. Additionally, NOXA is seen to degrade anti-apoptotic proteins MCL1 and A1, to further induce apoptosis [82]. The free pro-apoptotic proteins BAX and BAK translocate to the mitochondria (which is also stimulated through binding to PUMA), to induce mitochondrial outer membrane permeabilization (MOMP), leading to release of cytochrome c [74,81]. Secreted cytochrome c subsequently promotes the oligomerization of APAF1, which is also transcribed by P53, to initiate apoptosome formation [72,78]. Finally, different caspases will be recruited and activated through cleavage, including executioner caspases 3 and 7 to carry out apoptosis by cleaving all proteins in the cell [78,84].

## 2. Inhibitory Mechanisms of the P53 Pathway

As the P53 pathway is fundamental in maintaining genomic integrity in the face of cellular stress signals, it is unsurprising that approximately 50% of all solid cancers contain inactivating mutations within the *TP53* gene [50]. Additionally, it is hypothesized that nearly all cancers have a compromised P53 pathway, as the remaining 50% of cancers that lack *TP53* mutations often have mutated genes that lie either up- or downstream of P53 and may also lead to the inactivation of the P53 pathway [15,16]. In the following sections, different mutations and modifications of P53 and its main negative regulators Mouse Double Minute 2 and 4 (MDM2 and MDM4, respectively) will be described in further detail. Additionally, alternative mechanisms are also highlighted to provide a complete overview of the large variety of mechanisms that inactivate the p53 pathway.

### 2.1. TP53 Modifications

#### 2.1.1. TP53 Mutations

It has been estimated that the entire human genome contains approximately three million single nucleotide polymorphisms (SNPs), which suggests that an SNP occurs every 1000 nucleotides [85,86]. The *TP53* gene contains 19 kilobases, which suggests that it contains 19 SNPs; however, currently, 2060 *TP53* mutations have been identified in multiple different tumors and tumor cell lines, indicating a strong selection for mutations within this locus [87]. In fact, all 393 AA of the P53 protein are shown to be mutated in the International Agency of Research on Cancer I [66,87]. However, approximately 85% of all mutations, depending on the cancer type, are found within the DBD between residues 102 and 292 [66,87,88]. Approximately 10% of these DBD mutations are nonsense mutations that prevent the formation of the P53 protein, and another 80% of these mutations are missense [30,87]. Missense mutations are caused by the substitution of a single nucleotide within a gene, resulting in a different AA sequence while retaining the translation into a full-length protein. The extent to which *TP53* mutations contribute to tumorigenesis has been studied extensively. For example, many investigators have studied the temporal occurrence of *TP53* mutations; however, as many of these studies were performed retrospectively, no clear-cut conclusions were drawn [89,90]. Moreover, while the full-length protein is maintained, missense mutations are known to alter the structural conformation of the protein which may influence its binding affinity to proteins, DNA, or both [91]. In the context of *TP53*, biochemical analysis of different missense mutations demonstrated a partial loss of its DNA binding capacity, which may in turn affect its ability to transactivate target genes [92]. For example, the p53175P mutation has been shown to render P53 incapable of initiating apoptosis while remaining fully competent to contribute to cell cycle arrest [93]. 

Notably, approximately eight missense mutations account for 28% of all *TP53* DBD missense mutations, which are known as hotspot mutational sites [66,87,94,95,96]. This not only suggests a profound selection for mutations within the *TP53* locus but within specific sites of the DBD. Of note, in refractory GCTs, rare *TP53* mutations also appear to occur in the DBD [38]. An example of a hotspot mutations is an SNP at residue 175 where arginine is substituted by a histidine (R175H), which occurs in approximately 4–5% of all tumors and has been shown to impair the folding of the P53 protein [65,66]. This destabilizes the protein and interferes with its function as a tumor suppressor [92]. This mutation has also been shown to demonstrate a gain-of-function (GOF) phenotype, which indicates the acquisition of neomorphic functions that contribute to cellular transformation such as a disrupted cell cycle, invasiveness, and immortality [97,98,99]. It has been hypothesized that *TP53* missense mutations are selected for based on their impact on protein structure and subsequent oncogenic effects rather than being dependent on specific protein residues [98,100]. For instance, in glioblastoma, an infrequently occurring *TP53* mutation was identified that does not alter the protein structure [101]. However, this mutant was substituted in vitro for a frequently occurring oncogenic *TP53* missense that affects protein structure, and selection for the latter mutant was subsequently observed. 

*TP53* missense mutants have been shown to exert additional effects as well. For example, in some tumors, it was demonstrated that the mono-allelic R175H mutation was followed by the deletion of the second allele, causing a loss-of-heterozygosity (LOH) which in turn stabilized the R175H mutant and facilitated its GOF phenotype [102]. Additionally, this mutant was shown to exert a dominant negative effect in which the mutant protein sequesters WT protein during tetramerization and effectively inhibits the ability of WT protein to bind to DNA and engage its tumor-suppressive functions [51,52]. It has also been suggested that the dominant negative effect of P53 missense mutants is due to their higher propensity to form aggregates with the WT P53 protein but also with its homologs P63 and P73, disrupting their function [39]. These tumorigenic effects (i.e., LOH, GOF and a dominant negative effect) have been observed for multiple *TP53* missense mutants and remain an area of intense research [94,103,104,105]. Notably, P63 and P73 have also been implicated in GCTs (specifically testicular GCTs) [18,106,107,108]. For example, in contrast to germ cells, testicular GCTs did not express GTAp63, which has been attributed to epigenetic silencing, suggesting a possible mechanism that facilitates GCT development [106]. Moreover, while ΔNp73 was shown to inhibit NOXA expression in testicular GCTs after cisplatin exposure, the opposite was observed for TAp73 (also in the absence of P53), the latter may partially explain the propensity for GCTs to enter apoptosis in response to cisplatin [107]. Further information regarding the P53 homologs is summarized in Box 2.

Box 2 **Background information of the P53 homologs P63 and P73.**

The role of P53 (*TP53*) has been studied intensely; however, the P53 homologs P63 (*TP63*) and P73 (*TP73*) have also gained significant attention in the context of genomic integrity, embryogenisis, and cancer development since their discovery in the late 1990s [109,110,111,112,113,114,115]. These three proteins constitute the P53 family and share sequence homology most significantly within the TAD, DBD, and TD, having different chromosomal localizations [109,110,111,112,113,114,115,116]. Evidence suggests that *TP53* arose from an ancestral *TP63/73*-like gene throughout evolution [113,117,118]. Furthermore, two main protein variants of P63 and P73 have been identified caused by alternative promoter usage: the full-length protein (TAp63 and TAp73, respectively) and a protein product, lacking the TAD at the N-terminus, although they may retain transactivation activity through the presence of alternative TADs (ΔNp63 and ΔNp73, respectively) [113,114,115,116,119,120,121].

In short, the TA variant of P63 and P73 share the highest degree of sequence homology to P53 and have similar functions (depending on the isoform), including the transactivation of multiple P53 targets such as MDM2 (which in turn also negatively regulates P63 and P73), P21, PUMA, NOXA, and Bax, thus also playing a role in cell cycle arrest, DNA repair, and apoptosis [107,109,110,112,113,114,115,122,123,124,125]. For example, TAp763 and TAp63 have been implicated in DNA-damage induced apoptosis in oocytes, suggesting a role in maintaining the genomic integrity of the female germline [113,126,127,128]. Similarly, a more recently identified new P63 isoform, GTAp63, was found to be highly expressed in male germ cells, which induce apoptosis (including the transactivation of PUMA and NOXA) after cisplatin-induced DNA damage [106].

In contrast to the TA variants, the ΔN variants of P63 and P73 mostly act as dominant negative inhibitors of the transcriptionally active members of the P53 family including P53 itself, which highlights a complex degree of interaction between different members of the P53 family [113,114,115]. In addition, the TA and ΔN variants of P63 and P73 are also subjected to alternative mRNA splicing, generating multiple isoforms with overlapping and distinct functions that add to the diversity and complexity of the P53 family [113,114,115,116].

Moreover, P63 is known to have fundamental roles in epidermal development, whereas P73 is vital for neuronal development and maintenance, which is also evident from mouse studies where p63 or p73 knockout mice present with severe congenital defects in the respective organ systems (e.g., the skin and the brain) [113,129,130,131,132,133,134]. Moreover, P63 germline mutations have been associated with various syndromes, including ectodermal dysplasia [113,131].

In the context of tumorigenesis, the involvement of P63 and P73 has sparked considerable debate which is in part due to the low frequency of mutations, which contrasts the high frequency of P53 mutations in cancer [113,114,115,116,135]. However, it is generally accepted that the TA variants mainly function as tumor suppressors whereas the ΔN variants are mostly oncogenic and are often overexpressed in multiple cancer types; however, exceptions have been observed, and much remains to be investigated and confirmed [113,114,115,135,136].

#### 2.1.2. P53 Isoforms

The *TP53* gene consists of 13 exons that are subjected to alternative splicing [137]. This mechanism combined with two transcription start sites and an internal ribosomal entry site results in 12 possible isoforms of P53 [138,139]. Tumors harboring low rates of *TP53* mutations were demonstrated to a have higher expression of different P53 isoforms, which are also most often truncated at the N-terminus [139]. These isoforms lack the transcriptional function of the full-length P53 protein [140]. For example, Δ40P53 lacks the first transactivation domain, while the second TAD as well as the DNA binding domain are intact [141]. Moreover, this specific isoform is resistant to ubiquitination, rendering it more stable than the full-length protein [142]. Therefore, while Δ40P53 does not have transcriptional activity of its own, it can influence the full-length P53 protein by either repressing or activating it, depending on the cellular circumstances [142]. 

A second frequently occurring isoform is Δ133P53, where both TADs are missing, as well as the PRD and a part of the DBD [141]. It has been demonstrated that this isoform is capable of inactivating full-length P53 in a dominant negative manner, which may explain the observation that the expression of Δ133P53 represses apoptosis [139]. Additionally, due to a modified DBD, Δ133P53 induces the expression of different genes that contribute to tumorigenesis, including genes that promote angiogenesis and cellular reprogramming, which have been observed to be highly expressed in human glioblastoma cells [143]. On the other hand, in ovarian cancer, it has been shown that an increased expression of Δ133P53 correlates with improved survival and a lower change of recurrence [144]. Finally, another isoform that was recently discovered is Δ160P53, which lacks the same domains as Δ133P53 but lacks a larger segment of the DBD [138]. While there is still little known about the consequences of higher levels of Δ160P53, it was recently discovered that it is able to promote cellular invasion [145]. 

#### 2.1.3. P53 Post-Translational Modifications and Regulatory Elements

**Figure 6:**
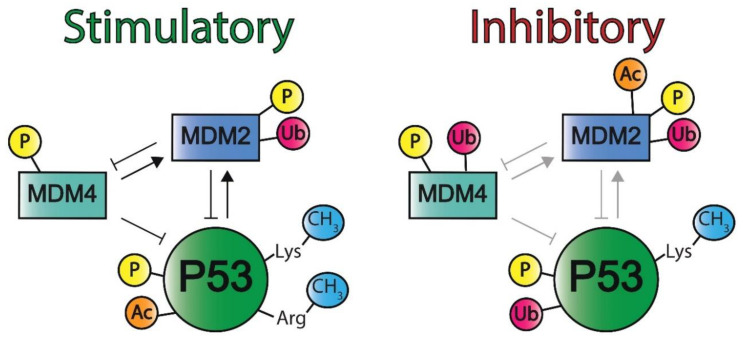
The different post-translational modifications of P53 and Mouse Double Minute 2 and 4. P53 can be activated (left) through phosphorylation (P), acetylation (Ac), and methylation (CH3) on lysine and arginine residues. Mouse Double Minute 2 (MDM2) can be phosphorylated and ubiquitinated (Ub) and Mouse Double Minute 4 (MDM4) can be phosphorylated. Post-translational modifications (PTMs) can also inhibit these proteins, which also decreases the regulation of each other (right). Phosphorylation, ubiquitination, and lysine methylation inactivate P53. Phosphorylation, acetylation, and ubiquitination inhibit MDM2, and phosphorylation and ubiquitination obstruct MDM4 activity. Of note, human genes and proteins are annotated in all capital letters (e.g., MDM2 and MDM2, respectively), whereas the murine homologs are annotated with a single capital letter (e.g., Mdm2 and Mdm2, respectively).

In addition to isoforms, the P53 protein is continuously subjected to a variety of post-translational modifications (PTMs) that are context-, tissue-, and cell-specific [146,147]. The most common PTMs are the methylation of arginine and lysine residues, phosphorylation of serines and threonines residues, and ubiquitination, acetylation, and sumoylation of lysine residues. Specific PTM patterns are present under physiological circumstances to regulate P53 activity, and aberrant patterns may disrupt P53 function and contribute to tumorigenesis. Different PTMs of P53 and its main negative regulators MDM2 and MDM4 are depicted in Figure 6 [148,149]. Further information of these negative regulators is described in Section 2.2. 

It has been demonstrated that multiple PTMs are simultaneously required to influence P53 activity, and crosstalk between these modifications has been observed [150]. For example, modifications can block each other and thus determine the activity of P53 combined [151]. 

Although little is known about the exact effects of P53 methylation, several enzymes have been characterized as either mono- or dimethylate lysine residues [152,153]. On one hand, methylation has been shown to activate P53 by stabilizing the protein and induce transcriptional activity [153]. However, repression of the DNA binding activity of P53 has also been observed [152]. Notably, crosstalk between methylation sites has also been suggested, as stimulatory methylation of a first lysine residue was shown to block the inhibitory methylation of a second lysine residue [154]. 

The combination of which lysine residues are modified and the number of methyl groups that are added to each lysine ultimately determines P53 activity. Thus far, the methylation of arginine residues appears to be solely regulated by protein arginine methyltransferase 5 (PRMT5), which targets three residues within the TD [155]. The induction of DNA damage causes the translocation of PRMT5 toward P53 to modify the protein, resulting in cell cycle arrest and a DNA damage response [155]. These specific residues have been seen to be mutated in tumors and inhibit the tetrameric formation of P53 [148]. 

The phosphorylation of P53 mainly occurs at the N- and C-terminal, and most residues are phosphorylated upon cellular stress to activate P53 [156]. However, several threonine and serine residues are continuously phosphorylated that target P53 for degradation, which is especially important in the absence of cellular stress [157,158]. DNA damage either induces phosphorylation or dephosphorylation of specific residues and enables cell cycle arrest and DNA repair. Phosphorylation is mediated through multiple redundant enzymes, as the same AA can be phosphorylated by multiple kinases and a single kinase is able to phosphorylate different residues [159,160,161]. Mutations in P53 could potentially lead to increased phosphorylation sites, which have also been seen to contribute to tumorigenesis. For instance, CDK4 can phosphorylate P53 at SNP R249S, resulting in Myc activation [149]. 

Ubiquitination of lysine residues, including those in P53, is established by three enzymes: an E1 ubiquitin-activating enzyme, an E2 ubiquitin-conjugating enzyme, and an E3 ubiquitin-ligating enzyme [162]. An important E3 ubiquitin ligase that functions as one of the main negative regulators of P53 is MDM2, which can ubiquitinate P53 at six residues present in the C-terminus [163]. Mono-ubiquitination translocates P53 toward the cytoplasm, where it may inhibit autophagy by promoting apoptosis in a transcription-independent manner [164,165]. P53 poly-ubiquitinated by MDM2 will result in its degradation [166,167]. The importance of MDM2 in regulating P53 is illustrated by the numerous types of tumors harboring elevated levels of MDM2, resulting in increased degradation of WT P53 and thus contributing to tumorigenesis [166]. Moreover, MDM2 overexpression and TP53 mutations appear to be mutually exclusive, suggesting that both can independently and efficiently disrupt the P53 pathway and contribute to tumorigenesis [168,169,170]. 

Small Ubiquitin-like Modifier (SUMO) has a structural resemblance to ubiquitin, and both are attached to lysine residues via a comparable [171]. Sumoylation has been seen to promote P53 transcriptional activity, while it is not able to affect P53′s stability or localization [172]. 

Finally, amongst other proteins, P53 can be acetylated by histone acetyl transferases (HATs), which were initially discovered to be involved in epigenetic acetylation [173]. P53 acetylation was shown to occur at the C-terminus, which promotes its binding to its target genes [173]. Moreover, acetylation stabilizes P53, as most of the known acetylation residues can also be subjected to MDM2-mediated ubiquitination [150]. MDM2 is not able to form a complex with acetylated P53, thereby protecting acetylated P53 from degradation [174,175]. To balance the acetylation of P53, histone deacetylases (HDACs) come into play, which suppress the transcriptional activity of P53 [176]. Certain deacetylases such as SIRT1 have been found to be upregulated in tumors; however, it has also been seen to have a tumor-suppressive role in others [177,178]. This counterintuitively suggests that de-acetylation may also stabilize P53 despite it rendering P53 vulnerable to ubiquitination at the same residues and requires further investigation.

#### 2.1.4. Chromosomal Number Alterations

Many tumors present a mono-allelic *TP53* mutation and an additional loss of the remaining WT *TP53* allele [179]. Loss of the WT allele is known to result from its specific deletion or the duplication of the mutated allele. As mentioned before, it has been hypothesized that *TP53* missense mutations can drive the loss of the WT allele, which may be explained by the observations made with the R175H mutant in which the loss of the WT allele stabilized the mutant protein [50,98,99]. For example, in murine leukemia and lymphomas, it has been shown that this loss did not only cause the deletion of WT *TP53* but also of other genes that further exacerbated the tumorigenic phenotype [180]. In addition, the loss of the WT allele in these mice were found to be the result from a duplication of the mutated *TP53* allele rather than the deletion of the WT gene, a similar observation to an earlier performed database analysis [180,181]. Recently, it has been demonstrated that the loss of the WT P53 allele contributed to the tumor- and metastasis initiation with intestinal tumors in a mouse model [182]. This suggests that the loss of the WT allele contributes to tumorigenesis; however, the underlying mechanisms (i.e., mutant allele duplication or WT allele deletion) differ between cancers. 

### 2.2. Negative Regulators of the P53 Pathway

#### 2.2.1. MDM2 

MDM2 is an E3 ubiquitin ligase that functions as an important negative regulator of P53 (illustrated in Figure 6) and is often modified in tumors with WT *TP53* [62]. Its importance in regulating P53 is underlined by the observation that *Mdm2*-null mice resulted in embryonic lethality [183,184,185,186]. The MDM2 protein consists of 491 AAs and contains multiple domains including an N-terminal P53 binding domain, an acidic domain, a zinc finger domain, and a C-terminal Really Interesting New Gene (RING) finger domain [187,188,189]. In addition, MDM2 contains nuclear localization and export sequences [190]. MDM2 can regulate P53 through mono- and polyubiquitination. Firstly, facilitated by its p53-binding domain, RING-finger domain, and nuclear localization sequences, MDM2 binds at the p53 TAD and subsequently monoubiquitinates specific lysine residues of the p53 REG domain, which translocates p53 from the nucleus to the cytosol and inhibits its functions as a transcription factor [163,166,167,190,191,192,193,194]. Secondly, MDM2-mediated polyubiquitination of p53 leads to its proteasomal degradation [163,166,194,195,196]. Notably, MDM2-mediated P53 ubiquitination is also facilitated by the phosphorylation of specific serine residues within acidic domain of MDM2 that enable its interaction (and thus that of p53) with proteasomes [197]. Furthermore, mono- or polyubiquitination of P53 by MDM2 is suggested to be a result of the level of MDM2 in the nucleus [192]. Notably, a negative feedback loop has been identified to control P53 protein levels as high levels of P53 induced *MDM2* gene expression of which the protein product subsequently mediates P53 ubiquitination and degradation [198]. Finally, MDM2 also has P53-independent functions such as targeting E-cadherin for degradation through its ubiquitin ligase activity, which has been associated with increased cellular invasiveness [199]. 

#### 2.2.2. Elevated Levels of MDM2

*MDM2* amplifications, observed in multiple cancers, have been shown to result in increased levels of its RNA and protein product [188]. Firstly, *MDM2* amplification affects the critical balance in P53 protein levels as MDM2-mediated P53 ubiquitination is increased, which has been observed in sarcoma amongst other tumor types [102,110]. Therefore, the therapeutic potential of disrupting the interaction between MDM2 and P53 has been intensely studied in tumors harboring an *MDM2* amplification, and small-molecule inhibitors such as Nutlin-3a demonstrate a successful reactivation of the P53-dependent apoptosis in osteosarcoma, colon cancer, and GCTs [28,41,200,201,202,203].

Regarding its P53-independent functions, lower levels of E-cadherin in combination with high expression of *MDM2* has been observed in breast and ovarian cancer [199,204]. Another P53-independent function of MDM2 is its ability to bind to RB, resulting in the expression of cell cycle promotors such as E2F from RB which has been shown to increase cellular proliferation [205]. 

Amplification is not the only cause of increased expression of *MDM2*. The frequently occurring SNP at nucleotide 309 (resulting in a T > G substitution), within the promotor of *MDM2*, leads to a higher affinity of transcription factor SP1 for the *MDM2* promotor and thus increased *MDM2* expression [206]. This mutation has also been correlated to a high risk for colorectal and prostate cancer [207,208]. Especially, colorectal cancer has been extensively studied for this SNP, indicating a higher risk in Asian populations when one or both alleles are mutated [209]. The reason SNP309 has not been shown to correlate in other cancers is due to another SNP at nucleotide 285, which frequently occurs in combination with SNP309 [210]. This SNP285 counteracts the increased affinity of SP1 for the *MDM2* promotor and has been observed in breast and ovarian cancers [211]. The SNP285 has been shown to be more frequently occurring in citizens from countries as Norway, the Netherlands, and UK compared to Finland and China, possibly explaining the correlation between SNP309 and tumorigenesis in colorectal cancer [209,211]. 

#### 2.2.3. Isoforms of MDM2

As a result of alternative splicing, over 40 different isoforms of MDM2 have been identified in both tumor and healthy tissues, which often lack (part of) the P53 binding site [212]. For example, MDM2-B was shown to be expressed most frequently in tumors including ovarian and bladder cancers [213]. Moreover, Zheng and colleagues detected a higher level of mutant P53 when this specific isoform was expressed [214]. This may be explained by the observation that MDM2-B cannot bind P53 but is capable of binding and inhibiting the full-length MDM2 protein, thus inhibiting P53 degradation [215]. In the case of a co-occurring *TP53* mutation, this leads to stabilization of mutant P53 resulting in an increased tumor volume and metastasis [214]. Additionally, many other MDM2 isoforms either have a truncation at the C-terminus or the alternative splicing causes the C-terminus to be out-of-frame; however, these do not occur as frequently as MDM2-B, which complicates gaining a deeper understanding of its effect [212].

#### 2.2.4. Post-Translational Modifications of MDM2

Similar to P53, MDM2 is also subjected to PTMs, which regulate its downstream functions. Currently, three types of MDM2 PTMs have been identified, as illustrated in Figure 6. Firstly, MDM2 can be ubiquitinated, which includes auto-ubiquitination. In unstressed cells, this mechanism is responsible for maintaining a low concentration of MDM2 protein [216]. Although the auto-ubiquitination function of MDM2 is inhibited under stressed conditions, MDM2 degradation still occurs, suggesting that other E3 ubiquitin ligases can target MDM2 for degradation [217]. Notably, MDM2 auto-ubiquitination also stimulates MDM2 to bind to E2 ubiquitin-conjugating enzymes, which subsequently leads to increased P53 polyubiquitination and degradation [218]. Conversely, MDM2 de-ubiquitination is equally important and occurs through the ubiquitin hydrolase HAUSP, which inhibits MDM2 auto-ubiquitination and enables MDM2-mediated P53 degradation [219].

Secondly, MDM2 is subject to acetylation, which mostly occurs at two lysine residues: K466 and K467 [220]. Modification of these lysine residues, which are located within the RING-finger domain, was shown to be effectuated by histone acyltransferase CREB-binding protein (CBP) in vitro, which silenced the E3 ubiquitin ligase activity of MDM2 [220]. Moreover, these lysine residues are also located in the nucleolar localization signal; however, their acetylation did not affect MDM2 translocation toward the nucleolus [220]. Notably, the in vitro substitution of these lysine residues for glutamine resulted in diminished E2 ubiquitin ligase activity [220]. 

Thirdly, MDM2 serine residues can be phosphorylated and occurs at multiple residues through multiple different enzymes [221]. For instance, ATM kinase has already been mentioned for its role as a DNA damage sensor and inducing stabilization through phosphorylation of P53. However, murine studies demonstrated that it can also phosphorylate Mdm2 at serine 395 [222]. In a mouse model, Gannon and colleagues have shown that the phosphorylation of this residue weakens the ability of Mdm2 to ubiquitinate P53, leading to increased apoptosis [222]. In addition, AKT/PKB serine–threonine kinase has been observed to phosphorylate MDM2 at serine 166 and 188 [221]. After activation by PI3K, AKT-mediated phosphorylation promotes the translocation of MDM2 toward the nucleus, where MDM2 can target P53 for nuclear degradation [221]. *AKT* has been shown to be overexpressed in tumors, resulting in MDM2 phosphorylation and increased P53 degradation, thus contributing to tumorigenesis [223]. 

#### 2.2.5. Ribosomal Proteins Influencing MDM2

Additionally, other factors are involved in regulating MDM2 activity. One of these factors regards ribosomal biogenesis, i.e., the production and processing of RNA and proteins that form the 40S and 60S ribosomal compartments, which occurs within the nucleolus [224]. When this process is disrupted, ribosomal proteins may bind and inhibit MDM2, leading to P53 stabilization and cell cycle induction [225]. Multiple proteins are known to bind MDM2; however, these bind varying regions due to their differences in structure [226]. The negative regulation of MDM2 by ribosomal proteins contributes to the hypothesis that decreased ribosomal biogenesis (and thus decreased MDM2 inhibition) contributes to an increased cancer risk [227]. Conversely, high expression of ribosomal RNA and proteins has also been observed in cancer, which may be caused by concomitant increased oncogene expression and/or inhibition of tumor suppressors [228,229]. In malignant melanoma cell lines, the expression of different ribosomal proteins was higher compared to significantly less malignant cells [230]. In addition, ribosomal proteins mutations have also been linked to syndromes that predispose to cancer such as the 5q-syndrome (a subtype of myelodysplastic syndrome) [231]. 

#### 2.2.6. MDM4

MDM4 is a homolog of MDM2 and is structurally similar [187]. For instance, MDM4 also contains a P53 binding domain and binds P53 at its TAD, thus blocking its function as a transcription factor [101,143]. Additionally, MDM4 was shown to bind P53 via other domains, including its acidic domain [144]. Loss of *MDM4* has been shown to increase the expression of many pro-apoptotic P53 target genes, which underlines its relevance in inhibiting P53 [232]. Moreover, in mouse embryonic fibroblasts, Mdm4 was shown to block p300-induced P53 acetylation that normally stabilizes and activates the P53 protein, thus resulting in an inactivated protein [233]. In addition, similar to MDM2, MDM4 is fundamental to embryonic development in regulating P53-dependent apoptosis which was demonstrated by the embryonic lethality observed in MDM4- or MDM2-null mice [183,185,186,234,235]. Notably, MDM4 is detected at low levels in healthy adult tissues [236].

Several differences and non-overlapping functions have also been observed. Firstly, the loss of *Mdm2* could not be compensated by increased *Mdm4* expression and vice versa [237]. Moreover, MDM4 is a cytoplasmic protein that lacks nuclear localization sequences and therefore depends on MDM2 to be translocated to the nucleus [238]. In addition, the MDM4 RING finger domain lacks E3 ubiquitin ligase activity, which indicates that MDM4 cannot target P53 for degradation [239]. Nevertheless, MDM4 can bind and stabilize MDM2 through heterodimerization via the RING finger domains and thus indirectly contributes to P53 ubiquitination [239]. Conversely, it has also been suggested that this heterodimer is needed to degrade MDM4 and thus to stabilize P53 [240]. Additionally, MDM4 also has P53-independent actions, such as the inhibition of DNA damage repair mechanisms, which may result in genomic instability [241].

#### 2.2.7. Elevated Levels of MDM4

The overexpression of *MDM4* has been observed in many different tumors, adding up to 2.9% of all cancers according to The Cancer Genome Atlas using cBioPortal, with higher frequencies observed of retinoblastoma and breast cancer as well as tumor cell lines [233,242,243,244]. The exact mechanisms underlying MDM4 upregulation are not completely understood [245]. Nevertheless, increased *MDM4* expression due to amplification results in fast proliferating and immortal cells, as WT P53 is transcriptionally inactivated [233]. Moreover, p300-mediated P53 acetylation is also impaired when MDM4 levels are elevated. Notably, MDM4 has also been demonstrated to contribute to cell growth and tumorigenesis in the absence of P53 [246]. This is thought to be a result of the P53-independent functions of MDM4, such as the ability to induce genomic instability through the inhibition of DNA damage repair [241]. 

#### 2.2.8. SNPs and Overexpression of Transcriptional and Translational Regulators of MDM4

Estrogen receptor-α is an important transcription factor of *MDM4* and is frequently overexpressed in cancer [247]. In addition, cancer-related overexpression of K-RAS and IGF-I induce the expression of MDM4 and thus inhibit P53 [248]. 

Multiple SNPs within the *MDM4* locus have been correlated to increased tumor risk [249]. For instance, the rs4245739 polymorphism that results in an A > C substitution has been studied extensively and has been detected within ovarian, prostate, and breast cancer [250,251]. This SNP lies within the 3′ UTR of *MDM4* and was found to form a new binding site in the MDM4 RNA for microRNA-191 and 887 [250,251]. MicroRNAs (miRNAs) are non-coding molecules of approximately 22 nucleotides that bind to complementary mRNA strands, resulting in their subsequent silencing or degradation [252,253,254]. Regarding *MDM4,* miRNAs-191 and -887 inhibited *MDM4* translation, thus reducing its protein level and stabilizing P53. Increased expression of these miRNAs has been observed in prostate cancer patients [251]. Moreover, a significant decrease in survival among ovarian patients was observed in those harboring this SNP [250].

#### 2.2.9. Overexpression of MDM4-S

Many isoforms of MDM4 have been found and studied with nearly all being the result of alternative splicing [244]. For instance, MDM4-S is a transcript variant in which exon six has been skipped and therefore excluded from the final protein that targets it for nonsense-mediated mRNA decay, ultimately preventing the inactivation of P53 [245]. Additionally, in mice, this isoform was shown to correlate to decreased levels of full-length Mdm4, thus potentially increasing P53 levels and having a tumor suppressive effect [245]. This was additionally suggested to be caused by the decreased expression of the oncogene serine/arginine-rich splicing factor 3 (Srsf3), which facilitates the inclusion of exon six [245]. In a mouse model, the undifferentiated ES cells were shown to harbor high levels of Mdm4, which significantly decreased upon differentiation, and it was suggested that this resulted from increased exclusion of exon six and therefore increased Mdm4-S expression [245]. Moreover, the increased expression of Mdm4-S in melanoma appeared to reduce tumor growth, thus demonstrating its potential as a therapeutic target [245]. In contrast, other studies demonstrated that MDM4-S has an increased binding affinity for P53 compared to full-length MDM4, which may suggest increased P53 inhibition. For instance, decreased P53 levels as well as a higher level of MDM4-S relative to full-length MDM4 was detected in papillary thyroid carcinoma and soft tissue sarcoma [255,256], which in turn may also dispute both the tumor-suppressor function of MDM4-S and the oncogenic function of Srsf3.

Finally, Pant and colleagues observed increased MDM4-S expression in B-cell leukemia patients, however, they suggested that this isoform was a consequence of splicing defects in tumor cells rather than being a contributor to tumorigenesis [257]. These investigators also supported the notion that MDM4-S has the potential to become a biomarker for this cancer. 

#### 2.2.10. Post-Translational Modifications of MDM4

Similar to P53 and MDM2, MDM4 activity is also determined by the combination of PTMs, most notably phosphorylation and ubiquitination. For instance, the phosphorylation of tyrosine residue 99 (Y99), located within the P53 binding site, impairs the binding of MDM4 to P53, leading to P53 stabilization [258]. However, when this PTM is combined with the phosphorylation of tyrosine residue 55 (Y55) at the N-terminus, MDM4 is able to bind P53 and inhibit its function [258]. MDM4 can be phosphorylated by the DNA damage sensor ATM and its targets CHK1 and CHK2 [259,260]. The phosphorylation of serine residue 367 by CHK1 results in the translocation of MDM4 toward the cytoplasm, effectively inhibiting it from binding P53, whereas CHK2-mediated phosphorylation of serine residues 342 and 367 translocates MDM4 toward the nucleus [259]. Additionally, these phosphorylation sites are also recognized by MDM2 to mediate MDM4 ubiquitination and degradation [259]. P14^Arf^ also functions as a negative regulator of MDM4 by facilitating MDM2-mediated MDM4 ubiquitination [261]. In different cancers, it has been shown that MDM4 ubiquitination is inhibited by a ribosomal RNA, namely non-coding 5S rRNA, which binds to the RING-finger domain of MDM4 [262]. 

### 2.3. Alternative Mechanisms to Inactivate the P53 Pathway

#### 2.3.1. P21 

P21 (*CDKN1A*), also known as WAF1 and CIP1, is an important transcriptional target of P53; however, it is also regulated through P53-independent mechanisms [70,71,263,264,265]. The *CDKN1A* gene consists of 2118 base pairs with three exons and produces a protein of 164 AAs [70]. As mentioned before, *CDKN1A* contributes to P53-dependent cell cycle arrest and apoptosis, and therefore, its expression is increased as P53 levels rise in response to cellular stress signals such as DNA damage [266]. P21 is primarily known to function as a CDK inhibitor (CDKi) by binding to a variety of cyclin–CDK complexes [71]. These complexes are crucial for cell cycle progression, with specific CDKs–cyclin complexes involved in different cell cycle phases [267,268]. Upon DNA damage, P21 facilitates cell-cycle arrest through the inhibition of CDK2 [266]. In addition, P21 also directly inhibits DNA replication and repair by binding proliferating-cell nuclear antigen (PCNA), which an important processivity factor for these processes [172,173,174,269]. 

Moreover, during a cellular stress response, anti-apoptotic proteins such as BCL-2 are known to bind and sequester pro-apoptotic proteins BAX and BAK, which inhibits apoptosis and increases mitochondrial reactive oxygen species through activation of the PI3K–AKT–MMP2 pathway, ultimately increasing the invasive competence of the cell [270]. Similarly, P21 is known to interact with and inhibit multiple pro-apoptotic proteins in the cytoplasm, including multiple caspases [263,271,272,273,274]. However, paradoxically, P21 may also have pro-apoptotic functions, as it was demonstrated to complex with P53 and aid in sequestering anti-apoptotic proteins to form a trimer, which results in the release of pro-apoptotic proteins, leading to the induction of apoptosis and decreased ROS levels [275].

Furthermore, despite that P21 is not a transcription factor, it is capable of influencing the expression of a variety of genes [276]. For example, the inhibition of CDKs by P21 reduces the phosphorylation of and activates retinoblastoma proteins, leading to the inactivation of transcription factor E2F [277]. E2F functions as a transcription factor for a wide spectrum of genes, with functions including DNA repair, DNA damage checkpoint, and cell cycle progression [278]. 

While P21 has been researched extensively in relation to tumorigenesis, SNPs in this protein are rare, which complicates studying its effects [279]. Double knockout *Cdkn1a* mice did not show any developmental restrictions or increased tumorigenesis in the presence of WT P53 during the seven months in which they were studied [280]. However, Martín-Caballero and colleagues created similar homozygous *Cdkn1a*-null mice, which they studied for two years [281]. A large part of the cohort died due to complications of increased proliferation of T-memory lymphocytes; however, a significant portion of the mice developed tumors including lymphomas, carcinomas, and sarcomas [281]. Moreover, compared to WT P21 mice, P21-null mice had a prolonged survival after irradiation, which may be due decreased tumor growth caused by an enhanced apoptotic response [281]. In addition, the loss of *CDKN1A* was demonstrated to contribute to tumorigenesis when combined with the loss of other tumor suppressors or activated pro-oncogenes. For instance, inactivation of both P21 and the INK4 pathway, which also functions in CDK inactivation, results in enhanced proliferation and increased susceptibility of tumorigenesis [282]. Additionally, *Cdkn1a*-null mutant mice crossed into an oncogenic Ras mutant background showed increased and accelerated tumor growth compared to WT P21 [283]. Furthermore, the inactivation of P21 has also been observed through the methylation of its gene, which is mediated by growth factor independent 1 (Gfi1)v [284], which has been observed to be highly expressed in a variety of tumors [285,286].

Paradoxically, increased P21 levels have also been associated to cancer and predisposing syndromes [263,264,274]. For example, high levels of P21 have been detected in ataxia telangiectasia, which is an illness caused by mutations in the *ATM* gene with increased formation of lymphomas and leukemias, and P21 was demonstrated to contribute to tumorigenesis in Atm-null mouse fibroblasts [287]. The loss of *Cdkn1a* partially compensated for the senescent phenotype and increased the sensitivity of the cells toward irradiation, indicating that the high expression of *Cdkn1a* contributes to tumorigenesis. 

Increasing evidence is emerging that in contrast to the initial paradigm, P21 may not solely function as a tumor suppressor but also as an oncogenic protein (this has previously been reviewed in [263,272,274,288]). While these controversial functions have sparked considerable debate, it has been suggested that a plethora of factors including PTMs and its subcellular localization (nuclear versus cytoplasmic) may dictate the effects of P21, which may underlie why both its up- and downregulation is observed in different cancer types [263,273,274]. For example, it has been hypothesized that the nuclear localization of P21 is important for its tumor suppressor function, mostly through cell cycle arrest; however, when localized in the cytoplasm, P21 may function as an oncogene [263,273,274]. The oncogenic properties of P21 may firstly be related to its aforementioned anti-apoptotic functions that are associated to its cytoplasmic localization [263,272,274,289]. Furthermore, another mechanism that put P21 forward as an oncogene was demonstrated when in vitro high constitutive P21 expression resulted in the deregulation of replication license machinery, leading to replication stress and subsequent genomic instability [264,290]. Notably, this was observed in a P53-deficient background, which suggests that the P53 status of the cell also dictates whether P21 functions as an oncogene or tumor suppressor gene [264,265,274,290]. Of note, P21 is known to be regulated by both P53-dependent and -independent mechanisms [263]. It was later also demonstrated that in addition to P21-mediated genomic stability, P21 may also contribute to a shift toward error-prone DNA repair pathways (most notably the RAD52-dependent error-prone break-induced replication pathway), which further contributes to genomic instability [265].

#### 2.3.2. ARF

The INK4 locus encodes for multiple proteins including P16^INK4A^ from the *CDKN2A* gene (hereafter P16) and P14^ARF^ from the *ARF* gene (hereafter ARF) [291,292]. The latter was first discovered in mice when an alternative reading frame of the *P16^Ink4A^* gene in the INK4 locus of mice revealed the sequence of a second gene: *P19^Arf^*, which is the murine homolog of *ARF* sharing 50% sequence homology [293]. This suggests that this protein is not strongly conserved. The sequences encoding for human *P16* and *ARF* overlap substantially and share sharing the last two out three exons [292]. However, P16 is part of the INK4 family of which all members function as CDK inhibitors whereas ARF does not, which is also evident due to its structural differences [292]. Instead, ARF functions as a tumor suppressor by inactivating MDM2 and thus stabilizing P53, which explains how the loss of *ARF* can increase tumor susceptibility [294,295]. It has been shown that ARF is mainly localized in the nucleolus, where it restricts MDM2 from its function to degrade P53 [296,297]. In addition, ARF is also thought to neutralize the E3 ubiquitin ligase activity of MDM2, including MDM2, that is already bound to P53 [291,298]. A P53-independent role of murine P19^Arf^ was demonstrated in a P19^Arf−/−^ mouse that was crossed into a double Tp53^−/−^; Mdm2^−/−^ background, which also increased tumor susceptibility, and subsequent reintroduction of P19^Arf^ into these triple knockout mice resulted G1 cell cycle arrest [299,300]. This P53-independent function may partially explain the binding partners of P19^Arf^ [301]. One of these proteins is Aurora B, through which P19^Arf^ is thought to maintain chromosomal stability [302]. Furthermore, P19^Arf^ has been demonstrated to be involved in the development of the eyes and male germ cells and even the extra-embryonic structures, such as the yolk sac [303,304]. 

Inactivation of ARF has been observed in multiple tumors (also in a mutually exclusive manner with *TP53* mutations) [305]. For instance, approximately half of the malignant gliomas were shown to harbor an *ARF* deletion, either with or without the additional deletion of *P16* [305,306]. Of note, the deletion of the entire *CDKN2A* locus is not *ARF* specific and as P16 is also lost; however, deleterious mutations in exon 1β, which is the *ARF* promoter, will only inactivate ARF [307]. For example, in renal cell carcinoma and tumors in the intestinal system, ARF expression was specifically silenced through promotor methylation [307,308,309,310]. Moreover, in cancer cell lines with an unmethylated *ARF* promoter, MDM2 was found to be localized in the nucleus, whereas a colorectal cancer cell line with a hypermethylated *ARF* promoter revealed MDM2 to be localized in the cytoplasm as well [311]. Subsequent in vitro demethylation of the ARF promoter in this colorectal cancer cell line demonstrated a translocation of MDM2 from the cytoplasm toward the nucleus, where it was subsequently inactivated by ARF, leading to P53 activation. 

The overexpression of *ARF* was also found to contribute to tumorigenesis in a number of tumor cell lines, which is suggested to be caused by the initiation of autophagy, as both autophagy and tumor growth decreased when ARF was inhibited [312]. Xie and colleagues studied the metastatic capability of prostate cancer in double Pten^−/−^; Tp53^−/−^, knockout mice [313,314]. P19^Arf^ was shown to stabilize Slug, decreasing the expression of E-cadherin [313], and upregulate metallopeptidase 7 (MMP-7), which promotes the degradation of E-cadherin [314], both promoting epithelial–mesenchymal transition. With a similar double knockout background of Pten^−/−^; Tp53^−/−^, overexpression of P19^Arf^ was frequently demonstrated to be present in chemo-resistant bladder cancer [315]. 

SNPs and indels specifically affecting ARF have been detected in a variety of tumors. These can be present in exon 1β (the ARF promoter), which has been demonstrated in a human colon cancer cell line carrying a nucleotide deletion that results in a truncated protein [309]. In addition, a primary colon carcinoma was shown to have a point mutation at codon 12 in exon 1β, which was thought to impair the binding of ARF to MDM2 and subsequently MDM2 nuclear translocation [309]. In melanoma, five mutational sites were identified that were suggested to inhibit ARF activity [316]. Such mutations are thought to increase MDM2 levels and thus the degradation of P53, thus contrasting the function of WT ARF.

#### 2.3.3. TRIM

The tripartite motif (TRIM) proteins were discovered in 1992, long before their functions were elucidated [317,318]. These proteins mostly contain three zinc-finger domains, two B-boxes, a coiled-coil domain, and a RING-finger domain, which gives the protein E3 ubiquitin ligase activity [317]. Multiple TRIM proteins have been identified and most of them homodimerize through their coiled-coil domain [318,319]. As these proteins are overexpressed in many tumors they are hypothesized to contribute to tumorigenesis [320,321]. For instance, increased TRIM23 expression is correlated with a poor prognosis in colorectal cancer [321] and lung adenocarcinoma [322]. TRIM23 can bind to P53 through its RING-finger domain and mediate P53 ubiquitination and subsequent degradation [321]. In colorectal cancer, elevated TRIM23 levels were shown to correlate with tumor size and lymph node metastasis, which was suggested to be the result of impaired P53 signaling [321]. In lung adenocarcinoma, TRIM23 expression was shown to correlate with resistance to the chemotherapeutic agent cisplatin which was suggested to be the result of induced GLUT1/3 expression and inhibition of NF-κβ [322]. 

Additionally, TRIM32 was shown to be overexpressed in lung cancer cells lines and was found to decrease apoptosis cell-cycle arrest and senescence and enhance proliferation and invasiveness [323,324]. Moreover, in a study with multiple tumorigenic cell lines, P53 was found to regulate TRIM32 expression as the promoter of TRIM32 harbored a P53 responsive element [324]. 

Lastly, TRIM59 upregulation has been observed in gastric cancer, non-small cell lung cancer and osteosarcoma [320,325,326,327]. In gastric cancer, this upregulation resulted in a decreased expression of P53 targets including P21, suggesting the inactivation of the P53 pathway [320]. Moreover, TRIM59 overexpression was found to correlate with the metastatic capacity of gastric tumor cells and a negative impact on the 5-year survival rates of patients [320]. Additionally, in cholangiocarcinoma and colorectal cancer, TRIM59 was demonstrated to promote cellular proliferation [326,328]. In all cell lines used, the inhibition of TRIM59 resulted in decreased phosphorylation of both PI3K and AKT, indicating that TRIM59 promotes cellular migration through these proteins [326,328]. 

#### 2.3.4. Long Non-Coding RNAs

The majority of the genome consists of non-coding sequences, including long non-coding RNAs (lncRNAs), which are often longer than 200 nucleotides [329]. Most of the nearly 15,000 known lncRNAs are transcribed by RNA polymerase II, and these molecules are modified by 5′ capping and poly-adenylation [330,331]. The expression of lncRNAs can be regulated by different mechanisms including transcription factors and epigenetic mechanisms [332,333]. LncRNAs are able to affect chromatin structure, thereby regulating gene expression and determining development, epigenetic reprogramming, and cellular pluripotency [330,334,335]. 

The P53 pathway can be inactivated through elevated expression of several lncRNAs, which has been demonstrated for Antisense Non-coding RNA In the *INK4* Locus (ANRIL) in prostate cancer [336]. Similar to ARF and P16, ANRIL is located in the *INK4* locus and was found to partially overlap with two genes, namely *P15^INK4B^* and *ARF* [337], the latter of which is known to influence the P53 pathway [294]. In prostate cancer, ANRIL overexpression was shown to recruit CBX7 (a Polycomb Repressive Complex 1 protein) to the *INK4* locus to enhance methylation and decrease the expression of all genes contained within this locus, including ARF, which may suggest increased P53 degradation [336]. Conversely, decreased ANRIL expression due to SNPs in the corresponding gene was detected in melanoma and may contribute to tumorigenesis through its ability to downregulate *CDKN2B* expression [338]. 

Moreover, the lncRNA Maternally Expressed Gene 3 (*MEG3*) was also shown to bind and activate P53, which was demonstrated by increased activation of P53 target genes and decreased expression was detected in pituitary adenomas [231,236]. In mice, knockout of *Meg3* resulted in overexpression of *Vegf* and *Notch*, leading to increased angiogenesis [333]. This was explained by the earlier observation that P53 represses *VEGF* expression breast cancer [339]. 

#### 2.3.5. MicroRNA 

As previously mentioned, miRNAs are short sequences of approximately 22 nucleotides [252]. These are initially formed as long primary transcripts, which are cleaved by Drosha to process them toward stem-loop precursors of about 70 nucleotides known as pre-miRNAs [340]. These are subsequently cut into single-stranded, mature miRNAs by Dicer [341]. Double knockout of Dicer in mice caused lethality during early development, which was suggested to be caused by the loss of stem cells, thus underlining the importance of miRNAs during early embryonic development [342]. MiRNA can bind the 3′ UTR of the mRNA and contribute to post-transcriptional regulation [254,343]. In short, mature miRNAs are incorporated into and RNA-induced silencing complex (RISC), which enables this complex to target mRNA that is at least partially complementary to the miRNA [253,344]. If the targeted mRNA has a high degree of complementarity, it will be cleaved and degraded, whereas low complementarity results in the inhibition of its translation [254]. 

Several miRNAs that target P53 have been identified, including miRNA-125 [345]. This specific miRNA was shown to be upregulated in prostate cancer cells [346] and myeloid leukemia [347]. In human cells as well as zebrafish embryos, miRNA-125 was able to bind to the P53 mRNA and inhibit its translation, possibly contributing to tumorigenesis [345]. Moreover, in prostate cancer cells with elevated miRNA-125 levels, inhibition of P53 was shown to result in increased growth [346]. It was found that the mRNA coding for apoptotic protein BAK contained a binding site for miRNA-125, which was confirmed by decreased BAK protein levels after ectopic expression of miRNA-125 [346]. A similar mechanism was demonstrated for miRNA-24 in hepatocellular carcinoma, which was shown to target P53, and elevated miRNA-24 levels resulted in increased invasiveness [348]. 

Conversely, other miRNAs have been shown to increase P53 signaling through inhibition of P53 inhibitors such as MDM2 [349,350]. For example, the downregulation of miRNA-339-5p (hereafter miRNA-339) was observed in colorectal cancer [351] and breast cancer [352], and it was later demonstrated that this miRNA was able to bind the 3′ UTR of MDM2 mRNA in human colorectal cancer cells [349]. Both mRNA and protein levels of MDM2 are decreased when miRNA-339 is elevated, suggesting high complementarity. Moreover, through the activation of P53, miRNA-339 was demonstrated to suppress the migration and invasion of colorectal cancer cells [349]. Additionally, miRNA-1827 was also shown to bind MDM2 mRNA, and elevated expression resulted in senescence and apoptosis [350]. 

MiRNAs can also be present within a cluster in the genome such as the miRNA-371a, -372, -373, and -373* cluster. In human fibroblasts with overactivated RAS, elevated expression of miRNA-372 and -3 resulted in continued proliferation, which was more pronounced compared to *TP53* knockdown [56]. Moreover, miRNA 372 and -3 were found to be highly expressed in testicular GCTs and GCT cell lines that harbored WT *TP53* [56]. It was further hypothesized that miRNA-372 and 3 are able to inactivate P53 through translational inhibition of MDM2 inhibitory protein LATS2 [56]. It was also recently demonstrated that murine miRNA-291a-3p (the murine homolog for miRNA-371a) inhibited senescence in human dermal fibroblasts by targeting several components of the P53 pathway [353]. Moreover, the human miRNA-371a-3p demonstrated similar anti-senescence activity, suggesting that all miRNA members of this cluster may have similar functions involved in inactivating the P53 pathway [353]. In addition, this specific miRNA cluster may soon function as a biomarker for GCTs [57,58,59].

#### 2.3.6. CTCF

An important tumor suppressor that is involved in epigenetic regulation is CCCTC-binding factor (CTCF) [354]. In chickens, CTCF was first found to bind CCCTC-sites in the oncogene *c-myc* and repress its expression [355], which was later also demonstrated in mammalian cells [356]. CTCF has 11 zinc-fingers within its DNA binding domain [357]; however, these are not all involved during every DNA-binding event [358,359]. Rather, multiple combinations are possible due to this large number of zinc-fingers, which enables CTCF to bind to approximately 125,000 cell type-specific, either inter- or intragenic or at the promotor [360]. CTCF is also essential during embryonic development as CTCF-null are non-viable [361]. This protein requires several post-translational modifications to function, including sumoylation by SUMO proteins [362] and poly(ADP-ribosyl)ation by PARP [363]. 

The hemizygous deletion of CTCF, which occurs more frequently than homozygous deletion, increased the susceptibility of spontaneous and radiation and chemically induced tumorigenesis [364]. Loss of the locus that contains the *CTCF* gene is frequently detected in breast and prostate cancer, which underlines its tumor suppressor activity [365]. *TP53* is one of the genes with a CTCF binding site in its promotor, and CTCF binding has been shown to obstruct histone methylation, which maintains an open chromatin structure and enables *TP53* transcription [366]. The *INK4* locus is also regulated by CTCF, as loss of CTCF increased the methylation and decreased the expression of CDKi P16 [367]. It was further demonstrated that CTCF can bind and repress *c-myc* expression, which in addition to inhibiting P16 and enabling *TP53* expression, further demonstrates its tumor suppressive role. 

Additionally, CTCF has also been suggested to have oncogenic potential, as increased expression was related to tumorigenesis within several breast cancer cell lines and tumor samples [368]. It has been hypothesized that CTCF could enhance tumorigenesis through the inhibition of pro-apoptotic protein BAX, which was increased after CTCF knockdown in breast cancer cell lines [369]. P53-levels were also elevated after CTCF knockdown in the same breast cancer cell line, resulting in increased cell cycle arrest and apoptosis [370]. 

## 3. Summarizing Discussion

**Figure 7:**
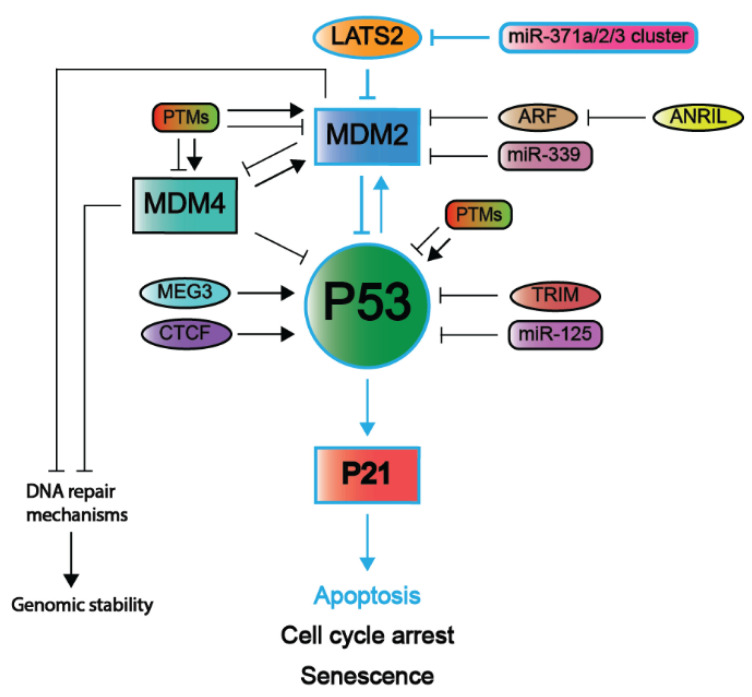
The regulatory mechanisms of the P53 pathway. P53 is regulated by MDM2 and MDM4, which are all affected by the PTMs and are also determining each other’s activity. MDM2 is indirectly activated by miR-cluster 371a-3 and ANRIL, while it is inhibited by ARF and miR-339. MEG3, CTCF, TRIM proteins, and miR-125 can regulate P53 activity directly. All these regulatory mechanisms combined will lead to the inhibition or activation of P21 expression, leading to apoptosis, cell cycle arrest, or senescence. MDM2 and MDM4 are both able to inhibit DNA repair mechanisms, resulting in genomic instability.

This review focuses on the P53 pathway as a guardian of the genome in cell types ranging from ES cells to somatic cells and describes different mechanisms that lead its inactivation and tumorigenesis (summarized in Figure 7). In response to cellular stress such as DNA damage, the P53 pathway is activated and initiates a plethora of downstream signaling pathways that ensure and maintain genomic integrity. For instance, P21 is a renowned downstream target that is involved in cell cycle arrest and cellular senescence [55,69,72]. In addition, autophagy and apoptosis can also be initiated to limit the damage [83]. It has been hypothesized that nearly all solid cancers harbor a disrupted P53 pathway, either directly through inactivating *TP53* mutations (occurring in roughly 50% of all tumors) or indirectly through alternative mechanisms such as described in this review [50,371]. An interesting exception to this pertains to GCTs in which *TP53* mutations rarely occur [33,35,36].

Regarding *TP53*, SNPs and other mutations and multiple isoforms have been demonstrated to increase tumor susceptibility. Moreover, multiple P53 isoforms resulting from alternative splicing have been identified, most of which are truncated at the N-terminus, and these have also been associated with tumorigenesis [139]. For instance, isoform is capable of binding and inactivating full-length WT P53 in a dominant-negative manner [57]. Moreover, due to a modified DBD, this isoform has been shown to transactivate oncogenes in human glioblastoma cells; however, this may be tumor-specific, as the opposite was observed in ovarian cancers where the expression of this isoform correlated to increased survival rates [143,144].

The activity of full-length P53 is also regulated by extensive PTM patterns, including methylation, phosphorylation, acetylation, and sumoylation, which are also known to engage in crosstalk, as shown in Figure 6 [150]. Whereas some *TP53* mutations have been found to obstruct multiple PTMs, other mutations may result in new modifiable residues [149]. These PTM patterns can also change by increased or decreased expression of the enzymes effectuating these modifications, which has been demonstrated by P53′s main E3 ubiquitin ligase MDM2 [191,199,204]. 

P53 activity is also strongly determined by its negative regulators such as MDM2. Elevated MDM2 levels have been shown to contribute to tumorigenesis through the increased degradation of P53 and tend to occur in a mutually exclusive manner with *TP53* mutations [168,169,170]. MDM2 levels can be increased through *MDM2* amplification but also through SNPs within its promoter [188,206]. For example, SNP309 is known to increase *MDM2* expression, as it enhances the affinity of transcription factor SP1 for the *MDM2* promoter. However, the effect of this SNP can be counteracted by the SNP285 SNP, which has been observed in ovarian and breast cancer [211]. SNP285 was demonstrated to be significantly higher expressed in Caucasians compared to Asian populations and may explain the stronger correlation between SNP309 and tumorigenesis in Asian populations [206,210]. However, a study within a Caucasian sample group showed a causative relation between SNP309 and earlier onset of prostate cancer [208]. Thus, further investigation is required to fully understand the co-occurrence of SNP309 and SNP285 in relation to cancer.

In addition, MDM2 activity is determined by a combination of PTMs, including phosphorylation and acetylation which, depending on the specific residues, either repress or enhance its activity [220,221]. Moreover, MDM2 can be degraded through ubiquitination and can also engage in auto-ubiquitination; however, the outcome of the latter remains controversial, as it has both been demonstrated to activate and degrade MDM2 [123,124,125]. It would be of interest to investigate whether the lysine residues subjected to auto-ubiquitination are similar to those subjected to ubiquitination by other enzymes. 

A final mechanism that determines MDM2 activity is ribosomal proteins, which bind and inhibit MDM2 in response to cellular stress signals [225]. It would be expected that decreased levels of these ribosomal proteins could contribute to tumorigenesis due to enhanced MDM2-mediated P53 degradation. However, several cancers demonstrate an increased level of ribosomal proteins [228,229]. This is likely confounded by the fast proliferation rate of cancer cells, which also requires high levels of ribosomal proteins [228]. However, a predisposition to tumorigenesis has been seen within conditions with a malfunctioning ribosomal protein modification [372]. These ribosomal proteins could be obstructed of their interaction with MDM2, resulting in the activation of P53. 

MDM4 is another important negative regulator of P53, and its overexpression has been observed in multiple cancers [233,242,243,244]. Its overexpression as well as increased expression of the transcription factors that regulate its expression is mostly caused by gene amplification. Similar to MDM2 and P53, MDM4 also has an isoform frequently occurring in tumors, Mdm4-S, where exon 6 is excluded from the protein [245]. It has been demonstrated to be highly expressed in B-cell leukemia, for which it may potentially serve as a biomarker [257]. Although Mdm4-S is prone to nonsense-mediated decay, its degradation is obstructed in B-lymphocytes, which leads to elevated levels of this isoform [257,373]. This has been suggested to be the result of increased *c-myc* expression, which is overexpressed through amplification in many tumors [373,374]. Conversely, other cancers have been shown to demonstrate higher levels of full-length Mdm4 compared to Mdm4-S, resulting in stronger P53 degradation [245]. This in turn could be the result of increased expression of the Srsf3 enzyme, which is responsible for the inclusion of exon 6 and was also demonstrated to be overexpressed in multiple tumors [375,376]. 

A final means to regulate MDM4 is through MDM2-mediated ubiquitination, which in turn is stimulated by the binding of ARF [261]. ARF is frequently lost in cancer, leading to the stabilization of MDM4 and thus the repression of P53 [261]. Loss of ARF also results in the activation of MDM2; however, it appears to obstruct MDM2-mediated P53 degradation, which decreases P53 levels [291,298]. Thus, ARF both appears to stimulate MDM2-mediated MDM4 ubiquitination (and thus P53 activation), yet it obstructs MDM2-mediated P53 ubiquitination [261,298]. This suggests that ARF facilitates the binding of MDM2 to MDM4, which reduces the number of free MDM2 protein that can bind and inhibit P53. This may be explained by the ARF binding site on the MDM2 protein. ARF binds to the N-terminal of MDM2, which is also required for P53 binding; however, the RING-finger domains in the C-terminus of MDM2 remain unobstructed, enabling MDM2 to bind to MDM4 [187,239]. Moreover, in contrast to a loss of ARF, overexpression of ARF has also been detected in tumors, which in mice and prostate cancer cells was demonstrated to increase the invasiveness of cells [313,314]. Notably, the murine study that associated P19^Arf^ overexpression with increased invasiveness was performed in a P53-null background and therefore does not conclusively demonstrate the tumor suppressive roles of P19^Arf^. Moreover, P19^Arf^ shares 50% sequence homology to human ARF, which further complicates their findings [292]. 

In addition to MDM2 and MDM4, another group of E3 ubiquitin ligase proteins known as tripartite motif proteins (TRIMs) were recently discovered to negatively regulate P53 [317,321]. TRIM23, TRIM32, and TRIM59 contribute to tumorigenesis when highly expressed, which is thought to be caused by inducing proliferation through multiple signaling pathways including the P53 pathway. For example, TRIM59 was overexpressed in gastric cancers and was suggested to inhibit the P53 pathway as the expression P53 targets including P21 was decreased [320]. In colorectal cancer and cholangiocarcinoma, TRIM59 was shown to promote proliferation, but this was thought to be through the PI3K/AKT or PI3K/AKT/mTOR signaling pathway, respectively [326,328]. It has been previously described that the PI3K/AKT pathway is able to induce P21 expression in ovarian carcinoma cells [377], which suggests that TRIM59 also has P53-independent functions.

Long non-coding RNAs are also known to regulate gene expression, and several may be indirectly involved in regulating the P53 pathway. For example, ANRIL and ARF are transcribed from the same locus, and the former was shown to decrease expression of the latter [337]. ANRIL-mediated inhibition of ARF subsequently results in higher levels of MDM2, which may lead to decreased P53 levels. This hypothesis was supported by the findings in prostate cancer where increased ANRIL levels resulted in lower activity of the P53 pathway [336]. Notably, the involvement of ANRIL in cancer may be context-dependent, as a specific SNP in melanoma was found to downregulate ANRIL expression, whereas a different ANRIL mutation was found to increase its expression in glioma [338]. Thus, the contributions of ANRIL to tumorigenesis are still debatable and require further attention. MEG3 is a second lncRNA that is often downregulated in cancer. In pituitary tumors and breast cancer, MEG3 downregulation was associated with a decrease in P53 activity [333,378]. 

Additionally, several miRNAs have been found to be overexpressed in tumors, which may disrupt the P53 pathway through P53 downregulation as well as through MDM2 overexpression [346,350]. For instance, P53 and the pro-apoptotic protein BAK contain a miRNA-125 binding site, which suggests that this miRNA has an anti-apoptotic function [346]. Additionally, in WT P53 expressing GCTs, it was demonstrated that MDM2 activity can be regulated by the miRNA 371-3 cluster by downstream inhibition of LATS2 (an MDM2 inhibitor) thereby indirectly inhibiting P53 [56]. 

The final P53 regulator discussed in this review is the CCCTF-binding factor CTCF which maintains an open chromatin structure at the *TP53* locus, thus enabling expression [366]. 

Further evidence regarding its tumor suppressive function was demonstrated by the discovery that CDKi *P16* is also under transcriptional control of CTCF [367]. *ARF* may also be regulated by CTCF, as it lies within the same locus as *P16*. Conversely, CTCF may also function as an oncogene, as CTCF knockdown resulted in an increased expression of P53 or the pro-apoptotic protein BAX in WT and mutant P53 breast cancer cell lines, respectively, which both increased apoptosis [369,370]. Moreover, CTCF is thought to repress *TP53* expression by inhibiting RNA polymerase II at the promotor [370]. However, it had been previously shown that CTCF interacts with RNA polymerase II to increase gene expression [379], which shows that the regulatory function of CTCF necessitates further investigation. In addition to the aforementioned mechanisms, it has been demonstrated that the P53 pathway may also be disrupted through aberrations of the P53 target P21. *CDKN1A* was initially proposed as a tumor suppressor gene for its role in cell cycle arrest. Therefore, it is unsurprising that decreased expression or loss of *CDKN1A* in combination with the loss of a second tumor suppressor gene or activated oncogene has been observed in multiple tumors [282,283]. However, as increased P21 levels have also been observed in multiple tumor types, there is growing evidence that P21 may also paradoxically function as an oncogene, which may be localization, cell type- and context-dependent, as well as related to the P53 status [263,264,265,274]. 

The main functions of these regulators have been elucidated; however, important questions remain to be answered to gain a complete understanding of their underlying mechanisms and their potential contribution to tumorigenesis. For instance, MDM2 contains two frequently occurring SNPs: SNP309, which increases its expression, and SNP285, which compensates for the effects of the former SNP [210]. The frequency of these SNPs differs among Asian and Caucasian populations, and further investigation into their individual and combined effects could provide further insights into their contribution to tumorigenesis. Furthermore, the involvement of the MDM4 isoform MDM4-S also provokes unanswered questions. On one hand, high MDM4-S levels are thought to be a result of *c-myc* overexpression followed by blocked nonsense-mediated decay [257,374]. In contrast, other tumors demonstrate upregulation of *Srsf3*, resulting in more Mdm4 compared to Mdm4-S [245,375]. Therefore, a deeper understanding of MDM4-S regulation is required to understand its contribution to tumorigenesis. Additionally, ARF-mediated MDM2 regulation is still largely unclear, as MDM4 is still ubiquitinated by ARF-bound MDM2, whereas MDM2-mediated P53 degradation is inhibited. In addition, lncRNAs have been shown to contribute to tumorigenesis; however, the exact pathways through which they elicit this effect remain debatable, as was demonstrated for MEG3 [380]. Finally, CTCF is thought to keep an open chromatin structure at the *TP53* locus and stimulate RNA Polymerase II required for gene transcription [366,379]. However, knockdown of CTCF has also been shown to increase P53 expression [369,370]. As a final note, it is most likely that the combination of the regulatory proteins and molecules are required to determine the outcome of P53 pathway activity and that multiple, as depicted in Figure 7. Notably, as an abundance of mechanisms that deregulate the P53 pathway has been observed in both somatic cells and cancers, including P53 mutations, many studies are dedicated to identifying novel targets that can restore this pathway and function as potential new cancer treatments. An overview of ongoing clinical trials regarding these types of approaches is summarized in Table 1.

In light of ES and somatic cells, it is evident that the P53 pathway is integral for ensuring genomic integrity; however, the subsequent downstream effects of this pathway are differentially regulated between these cell types. As mentioned before, somatic cells employ the P53 pathway to first induce cell cycle arrest and a DNA damage response (both error-free and -prone) to remain viable with the potential risk of accumulating harmful mutations, and they only initiate P53-mediated apoptosis when the damage cannot be repaired sufficiently [9,60,72]. In contrast, embryonic stem (ES) cells preferentially induce P53-mediated apoptosis as a failsafe mechanism to protect its multitude of cellular progeny and initiate a DNA damage response (mostly through error-free HR) only in a select few cases [9,10,23]. Similarly, GCTs contain many (epi)genetic and behavioral features that reflect their cells of origin, the ES cells and PGCs, and appear to initiate apoptosis in response to DNA damage, which is thought to explain their unique sensitivity to chemotherapeutics such as cisplatin [3,10,18]. To reiterate, teratomas are an exception to this rule, which is most likely due to their differentiated phenotype, rendering these tumors more comparable to somatic cells [3,15,16,17]. In GCTs, the preference to induce apoptosis has also been attributed to the P53 pathway, which as GCTs often have high WT *TP53* and *NOXA* expression concomitant with low expression of *CDKN1A* and other CDK inhibitors [18,21,23,24,25,26,27,28,29,33,34,35,36,37]. The low expression of other CDK inhibitors have also been observed in GCTs, which together with low P21 expression suggests a deregulated G1-S phase checkpoint reminiscent of ESCs, which are characterized by a short G1 phase [8,10,19,20,21,27]. Moreover, high *NOXA* and low *CDKN1A* expression may also be caused by the pluripotency marker OCT3/4, which is highly expressed in GCTs and their cells of origin and has been shown to augment the pro-apoptotic function of the P53 pathway through the indirect downregulation of *CDKN1A* and upregulation of *NOXA* [381,382]. Thus, these observations combined suggest that GCTs, similar to ES cells, may be biased toward P53-mediated apoptosis rather than attempting to repair the acquired damage.

In addition, despite the curative success of GCTs due to their sensitivity to chemotherapeutics such as cisplatin, GCTs may acquire resistance which, with the lack of targeted treatment, is inherently difficult to treat and exemplifies a significant shortcoming in current GCT treatment [383]. Thus, a significant proportion of GCT research aims to identify the underlying cause of GCT resistance, and it has been suggested that the deregulation of the P53 pathway may be a contributing factor [15,18,39,40,42]. Correspondingly, multiple mechanisms of P53 pathway deregulation have been observed in GCTs including rare *TP53* mutations and elevated P21, MDM2, and/or MDM4 levels, which have also been associated with resistance to cisplatin [22,27,34,37,39,384]. Furthermore, albeit that *TP53* mutations are rare in GCTs, an enlightening unbiased retrospective study of 180 GCTs was performed, and multiple P53 and MDM2 aberrations were observed in a subset of patients with aggressive and cisplatin-resistant GCTs [38]. This is further supported by the observation that disruption of the MDM2–p53 complex with a small molecule MDM2 inhibitor (Nutlin-3) resulted in the induction of P53 and increased cisplatin sensitivity in GCT cell lines that express WT *TP53* [28,41,203]. Additionally, as mentioned before, the miRNA-371a to -373* cluster has been found to be highly expressed in GCTs with WT P53 and have been shown to function as oncogenes by indirectly inhibiting P53 through the degradation of *LATS2*, which prevents it from inhibiting MDM2 [56]. Both this miRNA cluster and the elevated MDM2 and MDM4 levels suggest that while *TP53* mutations are rare, alternative mechanisms may be at play to deregulate the P53 pathway which may ultimately contribute to GCT cisplatin resistance. Furthermore, elevated P21 expression has also been associated with GCT cisplatin resistance; however, P21 appeared to be localized to the cytoplasm and was most likely regulated in a P53-independent manner [39]. It was further demonstrated that the cytoplasmic localization enabled P21 to inhibit apoptosis through protein–protein interactions with pro-apoptotic proteins, which again highlights that P21 may function as both an oncogene (or in this case contributes to the resistance phenotype) and a tumor suppressor which is most likely determined by many factors including subcellular localization [39,263,273,274]. Thus, whereas the P53 pathway may be deregulated, P21 expression may still be induced through P53-independent mechanisms with potentially oncogenic effects. Lastly, although not currently investigated in GCTs, elevated P21 expression levels also appear to dictate the DNA damage repair landscape, resulting in the use of error-prone DNA repair pathways that consequently contribute to genomic instability, and it would be of further interest to investigate this in GCTs [264,265]. It remains to be determined whether this also applies to GCTs, which, as mentioned before, are normally known to induce apoptosis rather than initiate DNA repair.

Nevertheless, the observations regarding the P53 pathway in GCTs thus far suggest that the transition from sensitive to (cisplatin)-resistant GCTs may be accompanied by the deregulation of the P53 pathway, enabling these cells to circumvent the characteristic preference for apoptosis and survive. This may additionally be exacerbated by the acquired ability to engage in cell cycle arrest and DNA repair. 

In summary, the activity of the P53 pathway is dictated by a myriad of regulating factors that form an elaborate and complex network. This review describes several of these factors and how they correlate to tumorigenesis through the disruption of the P53 pathway. Many aspects of these factors remain elusive and require further investigation to fully understand their contributions to tumorigenesis in general. Additionally, the involvement of the P53 pathway specifically in GCTs remains incompletely understood, and several of the mechanisms that regulate this pathway that have been described in this review may also be implicated in this specific context, which warrants further investigation. Moreover, due to the similarities between GCTs and ES cells, it may be of particular interest to further investigate the regulation of the P53 pathway in these cells and to further elucidate how this differs from somatic cells, which in turn may aid in understanding the involvement of this pathway in both the pathogenesis and acquired chemotherapeutic resistance in GCTs. Finally, further investigation into the complexity of the regulation of the P53 pathway in both ES and somatic cells may aid in the identification of new targets, the design of new studies, and ultimately new treatment options for multiple cancer types, including GCTs.

## Figures and Tables

**Table 1:** Ongoing clinical trials regarding novel targeting agents that modulate the P53 pathway.

Targeting Agents	Mechanism(s)	NCT Number
Phase I		
Vaccine therapy (i.e., gene therapy)	Virus-based adaption of *P53* expression in cancerous cells, mostly combined with chemotherapeutics	P53MVA: NCT02275039 Ad5CMVP53: NCT00004225
APR-246	A small molecule that binds mutant P53 and aids in folding to restore its wild-type conformation	NCT02098343
AMG-232 (KRT-232)	A small molecule that inhibits MDM2 and thereby activates P53	NCT03217266
ALRN-6924	A peptide that binds MDM2 and MDM4 to activate WT P53 in noncancerous cells, thus preventing side effects while enhancing chemotherapy effects in cancer cells.	NCT02264613
Phase I/II		
APG-115	MDM2 inhibitor that activates WT P53 with and without platinum-based chemotherapeutics	NCT03781986
